# The Symptoms Targeted for Monitoring in a Web-Based Tracking Tool by Caregivers of People With Dementia and Agitation: Cross-Sectional Study

**DOI:** 10.2196/13360

**Published:** 2019-06-28

**Authors:** Kenneth Rockwood, Myrlene Sanon Aigbogun, Justin Stanley, Helen Wong, Taylor Dunn, Chère A T Chapman, Susan E Howlett, Maia Miguelez, Lisa McGarrigle, Ross A Baker

**Affiliations:** 1 Geriatric Medicine Research Unit Halifax, NS Canada; 2 DGI Clinical Inc Halifax, NS Canada; 3 Division of Geriatric Medicine Dalhousie University Halifax, NS Canada; 4 Otsuka Pharmaceutical Development & Commercialization Inc Princeton, NJ United States; 5 Department of Pharmacology Dalhousie University Halifax, NS Canada; 6 Otsuka Canada Pharmaceutical Inc Saint-Laurent, QC Canada

**Keywords:** Alzheimer disease, dementia, agitation, neuropsychiatric symptoms, internet, caregiver

## Abstract

**Background:**

In people with dementia, neuropsychiatric symptoms (NPSs), especially agitation, are associated with worse quality of life and caregiver burden. As NPSs may vary with illness severity, knowledge of how people with dementia and their caregivers describe and rate the importance of agitation symptoms can improve the understanding of the clinical meaningfulness of the manifestations of agitation. The internet provides new opportunities to better understand patient experiences, as patients and caregivers increasingly look to Web-based platforms as a means of managing symptoms.

**Objective:**

The aim of this study was to examine Web-based reports from a dementia symptom website to better understand the symptoms of agitation and explore how they are being targeted for monitoring by caregivers of people with dementia.

**Methods:**

The Dementia Guide website hosts a Web-based database used by caregivers (97%) and people with dementia (3%).

From its 61 dementia symptoms, users can select relevant symptoms that they deem important to monitor or track the effects of treatment. We employed a staging algorithm to determine if individuals had mild cognitive impairment (MCI) or mild, moderate, or severe dementia. Agitation was defined using terms consistent with the International Psychogeriatrics Association’s provisional consensus definition. We compared the proportion of people with NPSs and agitation across stages of dementia severity and studied how many agitation-defining descriptors were selected, and how often they occurred, by stage.

**Results:**

As of March 2017, 4121 people had used the tracking tool, of whom 2577 provided sufficient data to allow disease severity staging. NPSs were tracked by 2127/2577 (82.54%) and agitation by 1898/2577 (73.65%). The proportion in whom agitation was tracked increased with increasing cognitive impairment: 68.5% (491/717) in people with MCI, and 72.50% (754/1040), 73.3% (378/516), and 90.5% (275/304) in mild, moderate, and severe dementia, respectively (χ^2^_3_=54.9; *P*<.001). The number of NPS and agitation descriptors selected also increased with severity (median number of NPSs=1, 2, 2, and 3 for MCI, mild, moderate, and severe dementia, respectively, Kruskal-Wallis H Test *H*_3_=250.47; *P*<.001; median number of agitation descriptors=1, 2, 3, and 4, *H*_3_=146.11; *P*<.001).

**Conclusions:**

NPSs and agitation are common targets for tracking over the course of dementia and appear more frequently with increasing disease severity. These common and distressing symptoms represent clinically meaningful targets in treating people with dementia.

## Introduction

### Background

Cognitive decline is the hallmark of dementia, but more often the accompanying neuropsychiatric symptoms (NPSs) trouble people with dementia and their caregivers the most. NPSs appear across all types and stages of dementia [1-3] in as many as 80% to 90% of people with Alzheimer disease (AD) [4,5]. Apathy, irritability, agitation, depression, and anxiety typically are noted most often [6]. When specific NPSs arise, they vary with the type of dementia and its severity. In AD, apathy and depression are most often seen early, whereas delusions, hallucinations, and aggression become more prevalent with disease progression [2]. In its various guises, agitation is common across all stages of dementia [2]. This likely reflects agitation’s multiple causes, including biological subsyndromes [7], reaction to declining cognitive function, and comorbid illnesses [8].

### Defining Agitation

The understanding of agitation continues to evolve. Recent work to evaluate NPSs in dementia has focused on the symptom of agitation. Without a commonly accepted consensus description of agitation, it has been difficult to compare studies or even to know which behaviors are included in studies of agitation [9]. For these reasons, the International Psychogeriatric Association proposed a provisional consensus clinical and research definition, so as to help define populations for clinical care and research [9]. The proposed definition has resulted in a liberally construed notion of agitation, extending even beyond commonly used *broad-spectrum* NPS measures, such as the Neuropsychiatric Inventory (NPI) [10] and NPI-Clinician Rating Scale (NPI-C) [11]. In consequence of the newer approach, it is important to know how bothersome agitation-defining symptoms might be to people living with dementia and to their caregivers. As more bothersome symptoms vary with illness severity, knowledge of how caregivers describe and rate the importance of agitation symptoms can improve the understanding of the clinical meaningfulness of the myriad manifestations of agitation. One useful method for eliciting this information is by patient-centered, individualized measures, which allow patients (crucially in dementia) and their caregivers to specify which symptoms they find most troubling [12,13].

### Web-Based Symptom Monitoring

The internet provides unprecedented access to the views of people living with chronic diseases. It is not yet clear as to how best their views can be elicited. Our group has pioneered one method of doing so. Since September 2006, the Dementia Guide website [14] offers a resource for persons with dementia (3% of users) and their caregivers (97% of users). Independently verified as credible using the DISCERN evaluation methodology [15], the site provides a mechanism for tracking symptoms important to caregivers and persons with dementia, thereby defining them as meaningful. This methodology is supported by previous work [16,17] and has allowed us to evaluate common but often understudied symptoms such as verbal repetition [18]. Although this approach cannot be used to estimate prevalence and incidence rates, which require representative population data, it can be used to compare frequencies in other nonrandom samples, and associations more generally, especially in relation to dementia severity.

### Study Objectives

Here we used the dementia SymptomGuide to better understand the symptom of agitation and how it is being targeted for monitoring by caregivers. Specifically, our objectives were to (1) define agitation using the dementia SymptomGuide and evaluate how often it was being monitored across the stages of dementia severity, (2) estimate the degree of agitation experienced by people with dementia and how it varied with stage, and (3) compare the reported frequency (episodes per day) of agitation to other NPSs.

## Methods

### Ethics

The Research Ethics Committee at Nova Scotia Health Authority provided approval for this study. Approval was sought from Nova Scotia Health Authority as one of the authors on this study (KR) is affiliated with the institution. SymptomGuide users consented to terms of use, which included allowing their data to be aggregated and used for research purposes. Users were assured that research findings would be presented in a manner that would not disclose personal or individual identifying information.

### Design, Participants, and Instrument

For this cross-sectional study, participants were recruited from the Dementia Guide website [14]. In addition to offering information, the website makes available the dementia SymptomGuide: a Web-based symptom tracker [19]. With the dementia SymptomGuide, individuals create a *Current Symptom Profile* to monitor the symptoms that are important to them. Users can choose from a standardized inventory of 61 symptoms and 609 symptom descriptions or enter symptoms and descriptions of their own. The predefined symptom descriptions overlap such that one symptom description can fall under more than one symptom. For example, the description *does not recognize current dwelling as home* from the *disorientation to place* symptom overlaps with *believes they live somewhere else* from *delusions and paranoia*. This redundancy is a built-in feature to allow users multiple ways to address common phenomena. Users note the occurrence of each selected symptom and record its frequency at baseline and whenever they choose to follow up. SymptomGuide users are also asked to rank their chosen symptoms from most to least important (from most to least bothersome). Symptom rankings were normalized as the rank divided by total number of symptoms reported resulting in a 0 to 1 scale, such that a weighted rank of 1 is most important. At baseline, SymptomGuide users are also asked to provide information on demographics, dementia diagnosis, and medication use. For those who select 3 or more symptoms, the dementia stage can be determined using a staging algorithm [17]. In this way, users have an individualized selection of symptoms that they track with standardized descriptions to allow comparisons across users. In this study, we used data from the users’ first visit (baseline) only.

We attempted to determine the level of cognitive impairment for all users with our staging algorithm [17]. Users who selected fewer than 3 symptoms were excluded from the analysis because the staging algorithm requires at least 3. Users for whom stage could be determined were categorized into 4 levels of cognitive impairment (mild cognitive impairment [MCI], mild dementia, moderate dementia, or severe dementia) as previously described by our group [17]. To control for outliers, if the number of symptoms selected by a user was greater than the 95th percentile (more than 23 symptoms), they were excluded from the analyses. Symptom frequencies were truncated at 10 episodes per hour (99th percentile) plus 1, such that symptom frequencies greater than 10 episodes per hour were imputed as 11 episodes per hour.

### Mapping Agitation to Symptom Descriptions

Agitation itself does not appear as one of the 61 symptoms in the dementia SymptomGuide Library. Instead, descriptions of many symptoms refer to agitation and its key components. To operationalize agitation, we took terms consistent with the International Psychogeriatric Association and NPI-C agitation definition [9,11] and mapped each term to SymptomGuide symptom descriptions. For example, the term *screaming* from the International Psychogeriatric Association’s consensus definition was mapped to the SymptomGuide descriptions: *yells, shouts, or screams*, *verbally attacks others (eg, shouts insults)*, *resists and refuses assistance with verbal outbursts*, and *yells at*, *or becomes easily angry with grandchildren*. In this way, 90 dementia SymptomGuide descriptions from 33 separate symptoms were combined to define agitation (Multimedia Appendix 1).

### Statistical Analysis

Participant characteristics were reported as frequencies and percentages, means and SD, or medians and ranges as appropriate. Percentages were based on the number of individuals with available data for each specific characteristic.

Objectives were evaluated using Pearson chi-squared test to compare the reported proportion of agitation and NPSs between stages of dementia severity (objective 1), Kruskal-Wallis one-way analysis of variance by ranks to compare the number and proportion of NPSs and agitation descriptors across groups, and a post hoc analysis with the Mann-Whitney-Wilcoxon test to determine pairwise associations (objective 2), and anticipating a non-normal distribution, we analyzed differences in reported episodes per day between symptoms using the nonparametric Mann-Whitney-Wilcoxon test (objective 3). All analyses were performed using R statistical software (R Core Team, Austria). All post hoc multiple comparisons were performed using Bonferroni corrections, by multiplying *P* values by the number of comparisons. Differences were considered statistically significant for values less than .05.

## Results

### Design and Participants

**Figure 1:**
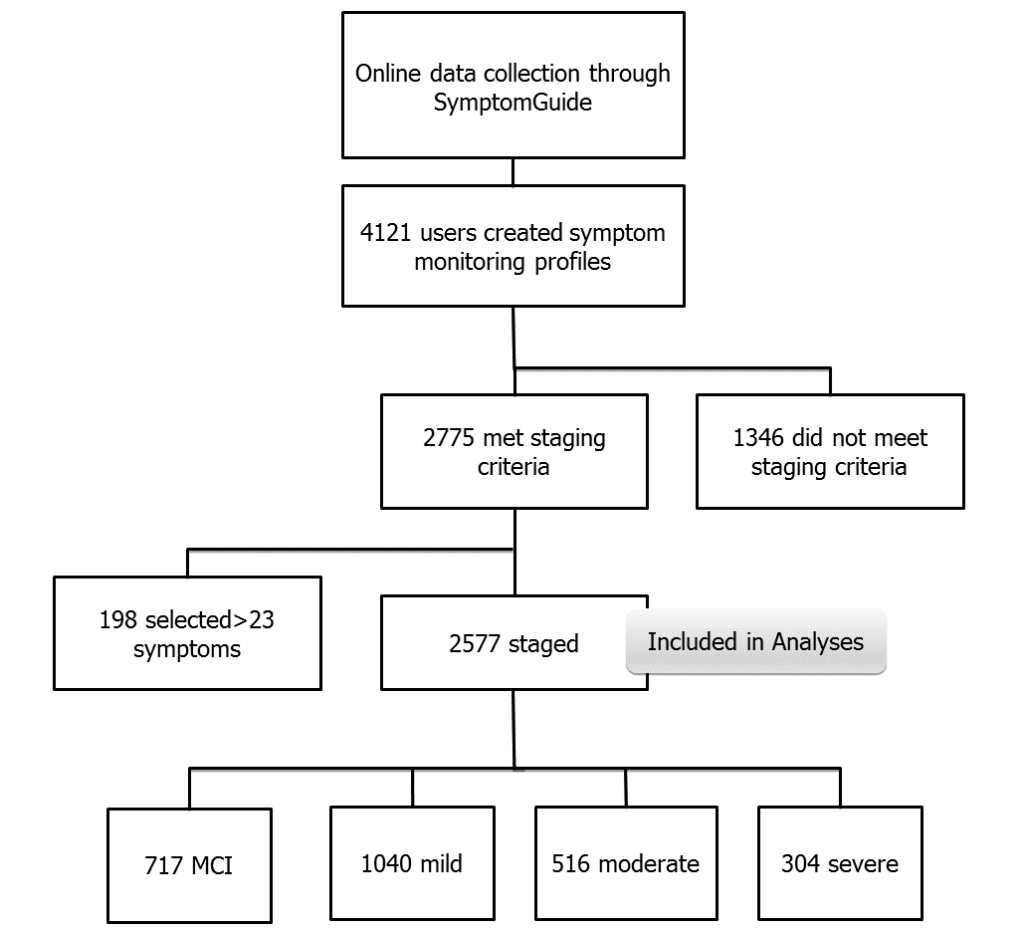
Participant inclusion chart. Participants were included in the analysis population if they met staging criteria excluding users who selected more than 23 symptoms (95th percentile). MCI: Mild Cognitive Impairment.

As of March 2017, the dementia SymptomGuide database consisted of 4121 participants who created a symptom profile to monitor symptoms as targets for treatment. Of those, 2577 had 3 or more symptoms needed for the staging algorithm and so were included in the study (Figure 1). Most participants were elderly North American women (Table 1).

**Table 1:** Participant characteristics.

Characteristic	Total	Mild cognitive impairment	Mild	Moderate	Severe
Participants, n	2577	717	1040	516	304
Age (years), mean (SD^a^)	76 (12)	74 (15)	75 (12)	77 (11)	77 (11)
Percentage^a^ of women	63	63	65	61	62
Percentage^a^ of participants with Alzheimer disease	61	100^b^	76	57	42
Percentage^a^ living in care facility^c^	13	11	7	20	19
Percentage^a^ with education ≥high school	77	82	78	78	71
Percentage^a^ of North Americans	87	84	87	89	86

^a^Statistic/percentage of participants who reported information.

^b^Two participants reported disease type in the mild cognitive impairment group.

^c^Care facility included retirement homes and nursing homes.

### Reported Symptoms of Agitation

**Figure 2:**
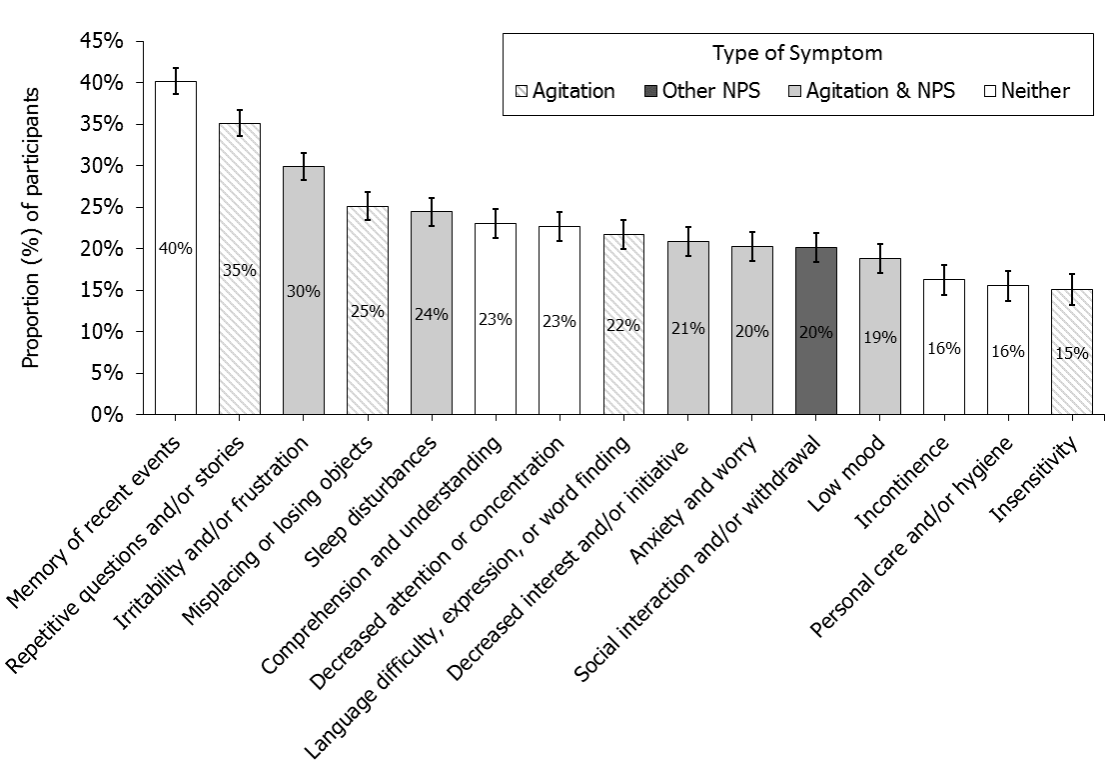
Prevalence of symptoms most frequently being monitored on the dementia SymptomGuide by type of symptom. The proportion (% of participants) ±SEp monitoring the 15 most frequently monitored symptoms. "Agitation"=non-NPS including a description of agitation, "Other NPS"=NPS which does not include a description of agitation, "Agitation & NPS"=NPS that includes a description of agitation, "Neither"=symptom is not an NPS and does not include a description of agitation. NPS: Neuropsychiatric Symptoms, SEp: Standard Error of Proportion.

Of the 61 symptoms, 33 contained descriptions of agitation, of which 15 were NPSs (Multimedia Appendix 2). For example, one description of agitation from a non-NPS (*looking after grandchildren*) is *yells at or becomes easily angry with grandchildren*. Two-thirds of the most common symptoms (10/15) were an NPS, included a description of agitation, or were both (Figure 2). On average, 7.7 (SD 4.7) symptoms were targeted (median=4, range 3-23) per participant. In most participants (82.54%, 2127/2577), at least 1 symptom was an NPS. Agitation was selected across all stages with 73.65% (1898/2577) of participants reporting at least one description of the symptom. Agitation was reported least often in the MCI group (68.5%, 491/717) and most often in the severe dementia group (90.5%, 275/304).

**Figure 3:**
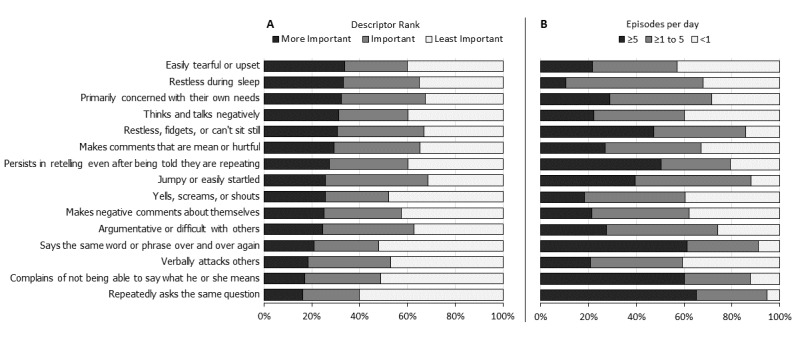
Ranking and daily frequency of the most common descriptions of agitation. Relationship between rank of importance (A) and number of episodes per day (B) of the 15 most commonly reported descriptions of agitation. “More Important” rank=1.00-0.67, “Important” rank=0.66-0.34, “Less Important” rank=0.33-0.00.

Agitation was most often described by verbalizations such as *Argumentative or difficult with others* (reported by 20.49%, 312/1523 of participants with ranked symptoms; Figure 3), *Makes comments that are mean or hurtful* (15.17%, 231/1523), *Yells, screams, or shouts* (8.93%, 136/1523), or *verbally attacks others* (8.54%, 130/1523). The 15 most commonly reported descriptions of agitation are shown in Figure 3. The frequency of the description did not correlate with the participants’ ranking of importance of the symptom (*r*=.03; *P*=.1). For example, the symptom descriptions *verbally attacks others* and *says the same word or phrase over and over again* were ranked similarly (mean 0.42 for both) but had significantly different reported median episodes per day (1.6 versus 5.0; *P*=.01). The reported occurrence of any description of agitation was, on average, 7 times per day (median 2, interquartile range: 1.0-5.0) which was significantly more frequent than other NPSs at 4 times per day (median 1, interquartile range: 0.7-4.0; *P*<.001). The rankings of NPSs were rated more important than those for descriptions of agitation (median weighted rank 0.62 vs 0.51; *P*<.001).

### Variations by Severity

**Figure 4:**
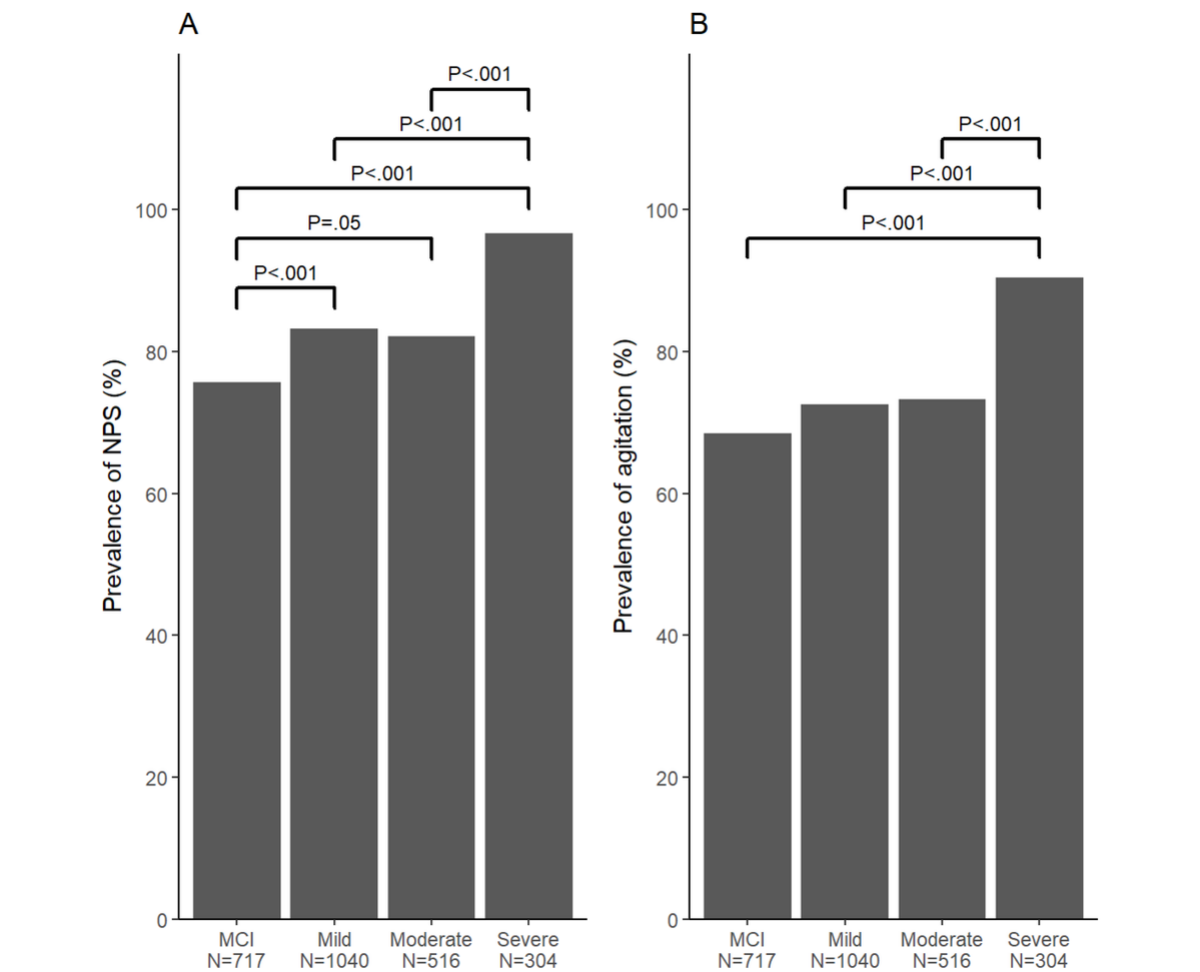
Proportion of participants who selected at least one NPS or description of agitation increased with stage. (A) % of participants who selected at least one NPS. (B) % of participants who selected at least one description of agitation. Bonferroni adjusted P values. MCI: Mild Cognitive Impairment, NPS: Neuropsychiatric Symptoms, p: P value.

**Figure 5:**
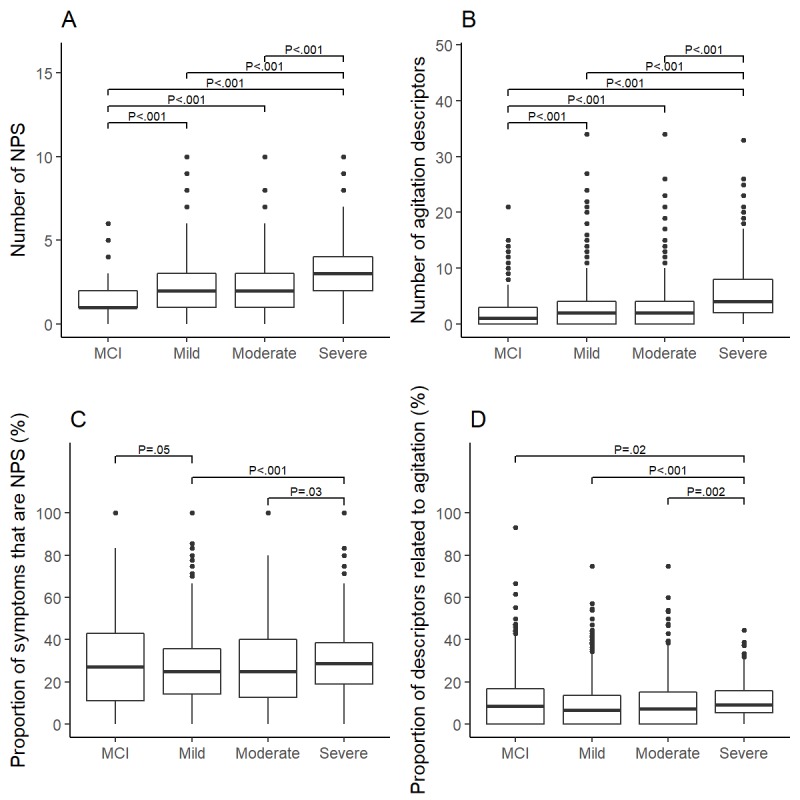
Number and proportion of symptoms that were NPS or descriptions of agitation were higher in the severe stage. Number of NPS (A) and descriptors of agitation (B) reported per subject in each stage. Proportion of symptoms targeted that are NPS (C) and symptom descriptions targeted that are descriptions of agitation (D) by stage. Outliers are shown here as observations above Q3 + 1.5 IQR (the 75th percentile + 1.5 times the interquartile range). Bonferroni adjusted P values. IQR: Interquartile Range, MCI: Mild Cognitive Impairment, NPS: Neuropsychiatric Symptoms, p: P value.

The proportion of participants with at least 1 NPS showed an increasing trend in relation to dementia severity—75.7% (543/717), 83.27% (866/1040), 82.2% (424/516), and 96.7% (294/304) for MCI, mild, moderate, and severe, respectively (χ^2^_3_=65.8; *P*<.001). Similarly, the proportion of participants with at least 1 agitation description increased with severity: 68.5% (491/717), 72.50% (754/1040), 73.3% (378/516), and 90.5% (275/304) for MCI, mild, moderate, and severe, respectively (χ^2^_3_=54.9; *P*<.001). A post hoc pairwise analysis showed that for people with severe dementia, there were high instances of both NPSs and agitation (Figure 4). We next compared the number of NPSs and agitation descriptions reported per participant by stage (Figure 5). There was a significant association between dementia severity and number of NPSs, with higher numbers of NPSs being reported in people with severe dementia (median 1, 2, 2, and 3 for MCI, mild, moderate, and severe, respectively; *H*_3_=250.47; *P*<.001). Similarly, there was a significant association between severity and agitation, with higher numbers of agitation descriptors being reported in those with severe dementia (median 1, 2, 3, and 4; *H*_3_=146.11; *P*<.001). For both measures, there were significant pairwise differences (*P*<.001) between every stage of dementia except mild and moderate. We also examined the prevalence of NPSs and agitation by stage and user-reported dementia diagnosis (Multimedia Appendix 3). The prevalence of both NPSs and agitation generally increased with dementia severity, in particular, in participants who reported a diagnosis of AD.

Finally, the proportion of total symptoms that were neuropsychiatric (number of NPSs divided by total number of symptoms) and the proportion of total descriptions related to agitation (number of agitation descriptions divided by total number of descriptions) were calculated for each participant (Figure 5). The proportion of NPSs varied significantly with stage, with the highest proportion occurring in those with severe dementia: median 27.3%, 25.0%, 25.0%, and 28.6% for MCI, mild, moderate, and severe, respectively (*H*_3_=17.39; *P*<.001). Similarly, the proportion of agitation description was highest in those with severe dementia: median proportion 8.3%, 6.7%, 7.2%, and 9.2% (*H*_3_=24.75; *P*<.001). Post hoc comparisons between stages revealed significant differences between the severe group and mild and moderate groups (*P*<.001 and *P*=.03, respectively) for NPSs and between the severe group and the MCI, mild, and moderate groups (*P*=.02, *P*<.001, and *P*=.002, respectively) for agitation. In participants who reported a specific dementia diagnosis, the number of NPSs and agitation descriptors selected increased with disease severity, significantly so in AD, vascular dementia, and frontotemporal dementia (Multimedia Appendix 4).

## Discussion

### Principal Findings

Using the SymptomGuide Web-based tracking tool, we determined how dementia caregivers selected symptoms of agitation as targets for monitoring. Across all stages, symptoms that define agitation, including NPSs, were tracked by most users. The proportion in whom agitation was tracked increased with severity of cognitive impairment. Although most of the symptoms that were chosen for monitoring tended to occur frequently, a few were tracked even when they occurred uncommonly. This suggests that the impact of a given symptom to caregiver distress might reflect either its content or its frequency or both. The range of specific symptoms that were identified for tracking also suggests the merit of a broad-spectrum approach to agitation, and perhaps the need for individualization to make tracking feasible. As it is important to validate our methodological approach, we compared our estimates of frequency of individual NPSs and agitation symptoms (from Web-based patient/caregiver reports) to data collected more traditionally in memory clinics. A large, nonrandom study by Siafarikas et al [20] used data from the Norwegian national registry of memory clinics (n=4571) to examine the frequency of NPSs and NPS subgroups in MCI and the different stages of AD. The study thereby notably provides a recent and contemporary database of symptoms and associations, collected to the high standard inherent in a national registry. We were interested to know whether trends similar to those found there could be detected using our Web-based data of predominantly North American caregiver reports. Recognizing that the differences in care and treatment practices between Norway and North America may be in play, nevertheless, our approach appears to be valid. For example, our estimate of the proportion of people with at least 1 NPS (82.54%, 2127/2577) corresponds closely to Norwegian estimates that NPSs were present in 87.2% of people with MCI or AD examined with validated neuropsychiatric cognitive assessments. Our data were consistent with that paper’s report of at least 1 NPS in 79.5% of MCI subjects and in 91.2% of those with AD and showed a similar trend of increasing NPSs with increasing dementia severity (Figure 4) [20]. In further support of our findings, Peters et al [21] characterized NPSs using data from A Canadian Cohort Study of Cognitive Impairment and Related Dementias [22] and found similar trends, with 74% of those that were cognitively impaired-not demented and 89% of those with dementia reporting at least 1 NPS. In keeping with our findings, they also found that a greater proportion of subjects experienced agitation with increasing levels of cognitive impairment (23% in the CIND group vs 36% in the dementia group). Similar comparability was seen when we mapped the dementia SymptomGuide symptom descriptions to the Neuropsychiatric Inventory-Questionnaire (NPI-Q) agitation/aggression domain used in the Siafarikas et al [20] study. Note that in contrast to the 90 SymptomGuide descriptions that aligned with what was described in the International Psychogeriatric Association’s provisional consensus report, only 50 such descriptions could be mapped to the NPI-Q agitation/aggression domain. However, the proportion of people with NPI-Q aggression/agitation increased with the severity of cognitive impairment—from 23% in people with MCI to 35% in people with moderate-severe dementia, close to our estimates using just those 50 descriptions: 19.0% (136/717) and 40.2% (330/820), respectively.

The similarity of our data with estimates from clinically adjudicated reports adds to our understanding of the validity and fidelity of Web-based data, at least where the Web-based reporting tool has been optimized for motivated users [19]. Resources such as the SymptomGuide increase the opportunity to capture the lived experience of people with dementia. In being able to demonstrate which of the symptoms with agitation are sufficiently salient to be monitored by caregivers, our data join those from the Norwegian registry report to offer empirical insights into the International Psychogeriatric Association’s provisional consensus clinical and research definition.

We were also able to assess the degree to which individuals experienced NPSs and agitation. We found that the number of NPSs and agitation descriptions reported per participant increased with dementia severity and were significantly different between all stages except mild and moderate. This finding should be interpreted carefully, however, as the total number of reported symptoms (and by extension, the total number of descriptions) also generally increases with severity. More telling measures were the proportion of a participant’s symptoms that were NPSs and the proportion of descriptions that related to agitation. We found similar estimates to those reported in other studies [20,21] showing that those with severe dementia experienced the highest degree of NPSs and agitation (median 28.6% of symptoms were NPSs; median 9.2% of descriptions were related to agitation). Of all the NPSs, agitation is often singled out as particularly troublesome and has become the focus of many investigations exploring the well-being of people with dementia and/or their caregivers [23,24]. To varying degrees of success, a range of pharmacological, psychological, and physical approaches are used to treat NPSs [1]. Despite their many advantages, instruments such as the NPI, NPI-Q, or NPI-C are highly structured and leave little room for customization or individualization by respondents. In other words, most self-report scales used for measuring NPSs contain predefined items or domains that have been deemed significant by the researcher, rather than the people with dementia and/or their caregivers. As a result, much less is known about the NPSs that are important to people with dementia or their caregivers, especially ones not included in NPS scales or measurements [9]. In this study, we did not have a comprehensive symptom inventory for each individual, however, we did have a list of symptoms selected for treatment, predominantly by caregivers. In this case, and as previously observed [25], agitation appears to be a potent symptom that is rarely ignored. The items selected by our caregiver/patient users were comparable in frequency and severity with those described by caregivers in the Norwegian national dementia registry, which also offers a comprehensive but nonrandom sample.

### Limitations

Our data must be interpreted with caution. As the dementia SymptomGuide does not include a symptom for agitation, we used 90 of 609 specific symptom descriptions to define agitation. This wide range of symptoms is in keeping with what has been proposed in the provisional consensus definition of the International Psychogeriatric Association [9]. Arguably, focusing on descriptors of behavior versus interpretation of behaviors is a strength, particularly given that the participants are nonprofessionals. However, such a broad definition of agitation may over represent the number of cases in this sample. For example, in keeping with that proposal, we included verbal repetition in our definition of agitation. This also reflects observations laid out elsewhere that verbal repetition might reflect anxiety as much as impaired memory [18]. In this sense, verbal repetition could be considered a form of psychomotor agitation, a prospect that requires additional inquiry. Furthermore, we note that the data in this study consist in self-reported questionnaire responses, with nontechnical accounts completed by care providers and a few people with dementia. Even so, this approach is in line with the goal of developing clinically meaningful data [12]. In this study, only data from the first (baseline) Web visit were used. It would indeed be interesting to track the patterns of symptoms over time in future studies, as numbers accumulate to allow sufficient disaggregation of data. The introduction of mobile health apps for smartphones and tablets should accelerate data acquisition.

### Conclusions

The importance of agitation symptoms as a target for treatment in our study supports the need for more well-controlled studies of both nonpharmacologic and pharmacologic treatments of agitation across all stages of AD, as well as the need for a universally accepted operational definition of agitation in this context. In any era in which importance is being placed on the lived experience of people with dementia, how do we incorporate their point of view? How can we extricate their views from those of the people involved in their care? How can we balance the accessibility of Web-based resources with the interpretability of the data which might be generated on the Web? Can Web-based data be used to detect patterns in treatment response that might inform our understanding of mechanisms in dementia? These questions are motivating additional inquiries so that we can develop a more well-rounded understanding of how dementia affects the lives of the people to whom it is a challenge.

## Multimedia Appendix 1

Agitation descriptors.

## Multimedia Appendix 2

Dementia SymptomGuide symptom listing.

## Multimedia Appendix 3

Prevalence of neuropsychiatric symptom (NPS) and agitation increases with dementia stage, especially in Alzheimer disease. Proportion of participants who selected at least one NPS (A) or description of agitation (B), stratified by user-reported dementia type. Differences in proportions were tested in each dementia type by Pearson’s chi-squared test. Note that patients with MCI were not included in this analysis because they did not provide dementia diagnoses. Bonferroni adjusted P values.

## Multimedia Appendix 4

Number of neuropsychiatric symptom (NPS) and agitation descriptors increases with dementia stage in Alzheimer disease, vascular and frontotemporal dementia. The number of NPS (A) and descriptors of agitation (B) tracked per participant in each stage, stratified by reported dementia type. Outliers were omitted for improved clarity. For each type of dementia, differences in number were tested by Kruskal-Wallis one-way ANOVA by ranks. Note that patients with mild cognitive impairment were not included in this analysis because they did not provide dementia diagnoses. Bonferroni adjusted P values.

## Abbreviations

AD: Alzheimer disease
MCI: mild cognitive impairment
NPI: Neuropsychiatric Inventory
NPI-C: Neuropsychiatric Inventory-Clinician Rating Scale
NPI-Q: Neuropsychiatric Inventory-Questionnaire
NPS: neuropsychiatric symptom
