# Supplementary material for: The Symptoms Targeted for Monitoring in a Web-Based Tracking Tool by Caregivers of People With Dementia and Agitation: Cross-Sectional Study
Source: J Med Internet Res. 2019 Jun 28;21(6):e13360. doi: 10.2196/13360 (PMC6625216; doi:10.2196/13360)
Supplement: Supplementary file 1 [file jmir_v21i6e13360_app1.docx]

**Multimedia Appendix 1.** Agitation Descriptors

| **SymptomGuide**™ **Symptom Name** | **Description of Agitation** |
| --- | --- |
| Aggression | Yells, shouts, or screams |
|  | Physically attacks others (e.g. kicking, punching, or hitting) |
|  | Verbally attacks others (e.g. shouts insults) |
|  | Causes injury to themselves |
|  | Causes injury to others or to the caregiver |
|  | Physically or verbally threatens the caregiver or others |
|  | Resists and refuses assistance with verbal outbursts |
|  | Resists and refuses assistance with physical outbursts |
| Anxiety and Worry | Is jumpy or easily startled |
|  | Complains of bad nerves or a nervous stomach |
|  | Is restless, fidgets or can't sit still (e.g. bites nails or taps fingers on table) |
|  | Paces or wanders aimlessly |
| Appetite | Is not hungry or refuses to eat at mealtimes |
|  | Will eat food that is spoiled or rotten (doesn't recognize that food has gone bad) |
| Balance | Falls often |
| Bathing | Resists assistance with bathing or showering |
| Delusions and Paranoia | Believes that they live somewhere else (e.g. asks to go home) |
|  | Becomes jealous when attention is directed towards others |
| Disorientation to Place | Does not recognize current dwelling as home (e.g. asks to go home or packs to go home). |
| Eating | Refuses to swallow |
| Hobbies | Complains that hobbies or activities are tiring |
|  | Complains that hobbies or activities are too difficult |
| Inappropriate Language and Behaviour | Makes comments that are mean or hurtful (e.g. tells people they are fat or have a bad haircut) |
|  | Curses and uses foul language |
|  | Stands too close to people or touches them inappropriately |
|  | Makes sexually suggestive comments or advances |
|  | Physically or verbally threatens others |
|  | Spits, belches, or does other embarrassing things in social situations |
|  | Acts on previously controlled impulses (e.g. phones people in the middle of the night) |
|  | Reacts in a way that doesn't match the situation (e.g. laughs at a funeral) |
|  | Displays extreme emotional outbursts and over-reactions |
|  | Frequently makes unwarranted accusations towards others |
|  | Screams or shouts |
|  | Extreme emotional outbursts and over-reactions |
| Inappropriate Sexual Behaviour | Makes inappropriate remarks in public places |
|  | Says inappropriate things to people they know |
|  | Makes unwanted advances to people they know |
|  | Says inappropriate things to strangers |
|  | Makes unwanted advances to strangers (e.g. grabs or attempts to fondle) |
|  | Kisses or hugs more than would be appropriate to express affection |
|  | Attempts to have sexual intercourse with others |
| Insensitivity | Makes comments that are mean or hurtful |
|  | Reacts in a way that does not match the situation (e.g. laughs at a funeral) |
|  | Yells or shouts at others |
|  | Is primarily concerned with their own needs |
| Insight | Does not recognize potential danger or hazards (e.g. hot stove or broken glass) |
| Interest/Initiative | Complains of being bored; says there is nothing for them to do |
| Irritability/Frustration | Is argumentative or difficult with others |
| Language Difficulty/Expression/Word Finding | Complains of not being able to say what he or she means |
| Looking After Grandchildren | Yells at, or becomes easily angry with, grandchildren |
| Low Mood | Is easily tearful or upset |
|  | Has crying spells |
|  | Thinks and talks negatively (e.g. says you would be better off without me) |
|  | Makes negative comments about themselves; feels of little value |
| Misplacing or Losing Objects | Hides or hoards items |
| Obsessive Behaviour | Habitually hides items (e.g. money keys credit cards) |
|  | Hoards and hides food |
|  | Paces |
|  | Continuously wants to go to the same place (e.g. |
| Operating Gadgets/Appliances | Uses an appliance or tool for the wrong function (e.g. puts clothes in the dishwasher or uses cordless phone as a remote) |
|  | Uses an appliance or a tool improperly (e.g. moves vacuum side-to-side instead of back-and-forth) |
|  | Uses appliance or machinery in an unsafe manner (e.g. cannot control power saw) |
| Personality Changes | Has become self-centered |
|  | Complains and criticizes more |
|  | Becomes easily tearful or upset |
|  | Is always anxious or worried |
|  | Becomes scared or frightened easily |
| Physical Complaints | Complains of headaches |
| Reading | Complains that reading is difficult |
| Repetitive Behaviour | Engages in repetitive motions like rocking or tapping fingers |
|  | Always checks and rechecks things (e.g. that windows are locked or that the cat is in the house) or hoards items like money clothing or food |
| Repetitive Questions/ Stories | Persists in retelling stories or information even after being told he or she is repeating |
|  | Says the same word or phrase over and over again |
|  | Repeatedly asks same question |
| Restlessness | Fidgety and impatient in the late afternoon and early evening (called sundowning) |
|  | Wanders or paces around the home or outdoors |
|  | Has excess energy |
|  | Engages in repetitive motions (e.g. rocking or tapping) |
|  | Has difficulty falling asleep |
|  | Becomes distracted easily |
|  | Searches for things to occupy themselves with |
| Sensory Input | Places dangerous or inappropriate things in their mouth |
| Sleep Disturbances | Is restless during sleep (tosses and turns) |
| Spirituality and Religion | Refuses to dress appropriately for a religious service |
| Travel and Vacationing | Has a tendency to wander off in busy places (e.g. airports or bus stations) |
| Unsafe Actions | Eats or serves food that is spoiled or rotten |
| Wandering | Circles or paces around a room |
|  | Does not recognize current dwelling as home. |
|  | Appears restless or has energy to spare |
|  | Searching for something or someone in the past. |
