# Supplementary material for: The Symptoms Targeted for Monitoring in a Web-Based Tracking Tool by Caregivers of People With Dementia and Agitation: Cross-Sectional Study
Source: J Med Internet Res. 2019 Jun 28;21(6):e13360. doi: 10.2196/13360 (PMC6625216; doi:10.2196/13360)
Supplement: Supplementary file 2 [file jmir_v21i6e13360_app2.docx]

**Multimedia Appendix 2.** Dementia SymptomGuide™ symptom listing

| **Symptom Name** | **NPS** | **Agitation^a^** |
| --- | --- | --- |
| Activities with Other People |  |  |
| Aggression | X | X |
| Anxiety and Worry | X | X |
| Appetite | X | X |
| Attention/Concentration |  |  |
| Balance |  | X |
| Bathing |  | X |
| Comprehension/Understanding |  |  |
| Decision Making |  |  |
| Delusions and Paranoia | X | X |
| Disorientation to Place |  | X |
| Disorientation to Time |  |  |
| Dressing |  |  |
| Driving |  |  |
| Eating |  | X |
| Financial Management |  |  |
| Following Instructions |  |  |
| Hallucinations | X |  |
| Hobbies |  | X |
| Household Chores |  |  |
| Inappropriate Language and Behaviour | X | X |
| Inappropriate Sexual Behaviour | X | X |
| Incontinence |  |  |
| Independence |  |  |
| Insensitivity |  | X |
| Insight |  | X |
| Interaction with Friends and Family |  |  |
| Interaction with Strangers |  |  |
| Interest/Initiative | X | X |
| Irritability/Frustration | X | X |
| Judgment |  |  |
| Language Difficulty/Expression/Word Finding |  | X |
| Looking After Grandchildren |  | X |
| Low Mood | X | X |
| Low Self Esteem |  |  |
| Meal Preparation/Cooking |  |  |
| Memory for Names and Faces |  |  |
| Memory of Past Events |  |  |
| Memory of Recent Events |  |  |
| Misplacing or Losing Objects |  | X |
| Mobility |  |  |
| Obsessive Behaviour | X | X |
| Operating Gadgets/Appliances |  | X |
| Personal Care/Hygiene |  |  |
| Personality Changes |  | X |
| Physical Complaints |  | X |
| Reading |  | X |
| Repetitive Behaviour |  | X |
| Repetitive Questions/Stories |  | X |
| Restlessness | X | X |
| Self-Awareness |  |  |
| Sensory Input |  | X |
| Shopping |  |  |
| Sleep Disturbances | X | X |
| Social Interaction/Withdrawal | X |  |
| Spirituality and Religion |  | X |
| Telephone Use |  |  |
| Travel and Vacationing |  | X |
| Unsafe Actions |  | X |
| Wandering | X | X |
| Writing |  |  |
| ^a^Symptom includes a description of agitation;  NPS: Neuropsychiatric symptom | | |
