# Supplementary figures and images for: The Symptoms Targeted for Monitoring in a Web-Based Tracking Tool by Caregivers of People With Dementia and Agitation: Cross-Sectional Study
Source: J Med Internet Res. 2019 Jun 28;21(6):e13360. doi: 10.2196/13360 (PMC6625216; doi:10.2196/13360)

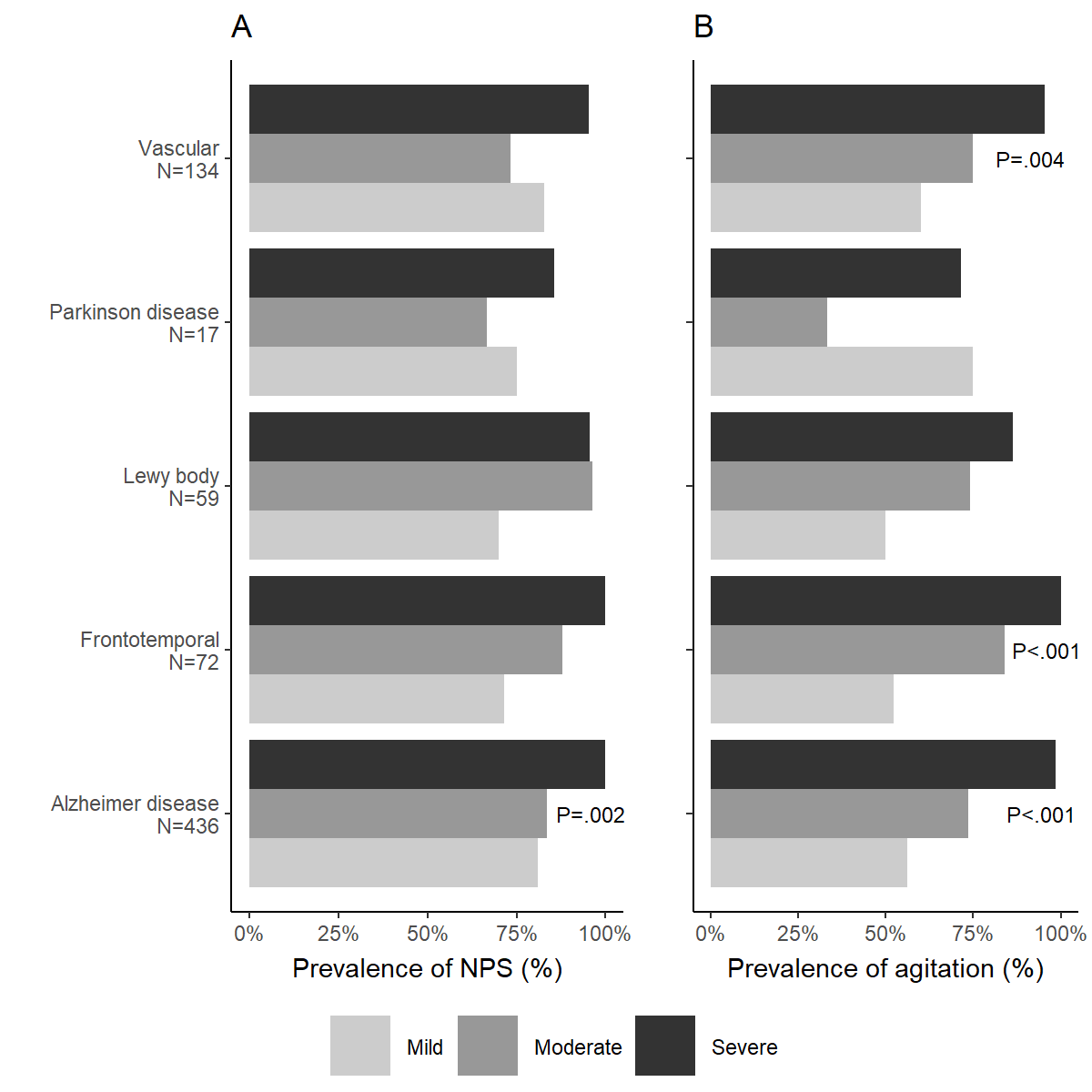

Supplement: Supplementary file 3 [file jmir_v21i6e13360_app3.png]

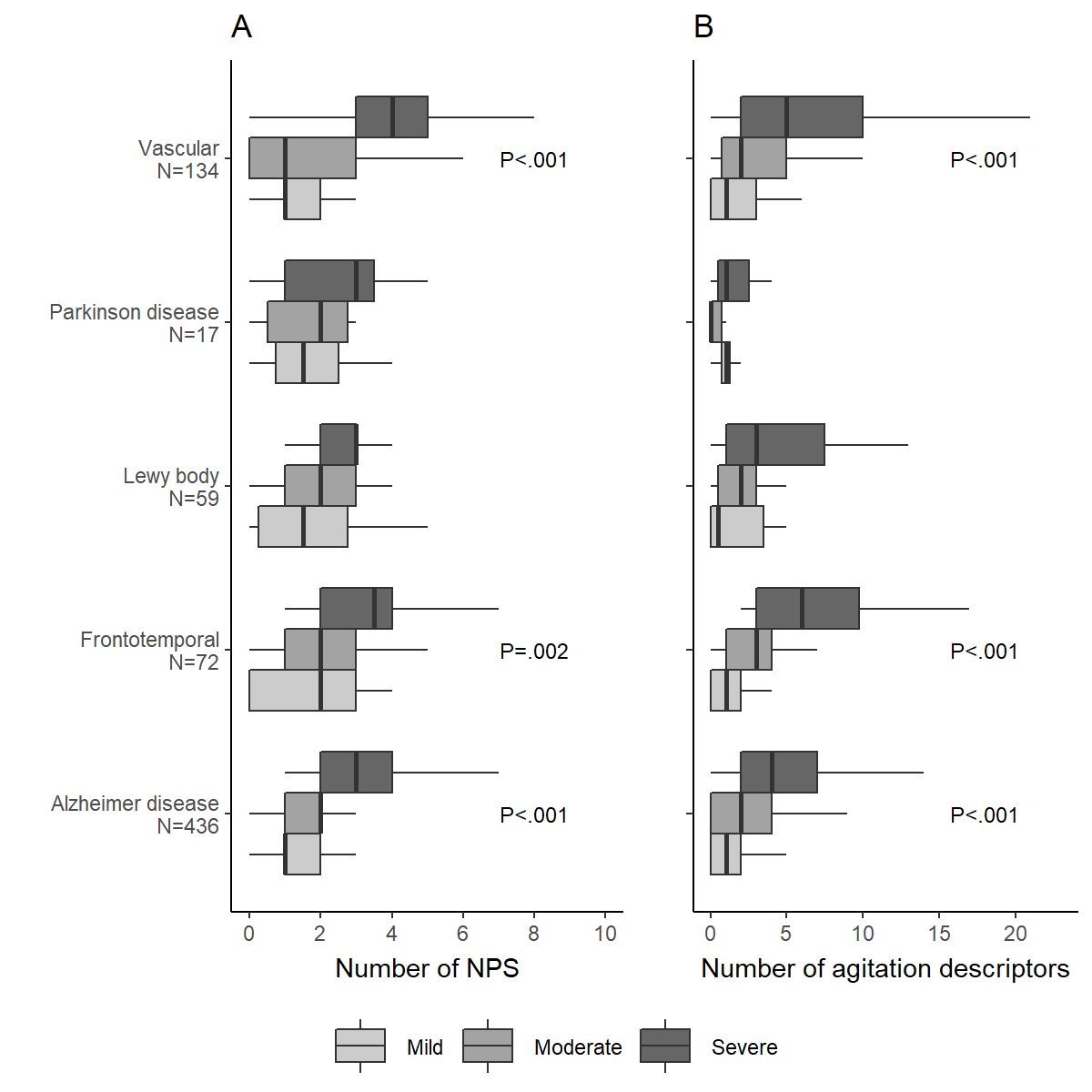

Supplement: Supplementary file 4 [file jmir_v21i6e13360_app4.png]
